# Puerperal Mania—with History of a Case

**Published:** 1881-12

**Authors:** John Macfarlan

**Affiliations:** Denver, Col.


### CLINICAL BUREAU.

---

### PUERPERAL MANIA—WITH HISTORY OF A CASE.

JOHN MACFARLAN, M. D., DENVER, COL.

In presenting this paper the object has been simply to incite discussion and an interchange of opinion upon a subject which, although noticed in the text-books, is treated of in a rather unsatisfactory manner so that but little aid is afforded by them in the actual treatment of the affection.

If, as the writer believes, it remains for homœopathy to make in the future the most rapid advances in the medical sciences, it becomes necessary for its advocates, in the investigation of disease and in attempting to arrive at just conclusions, to lay aside all personal preferences, all pet theories and to accept only such reasoning as is sustained by facts. And it is in the investigation of such diseases or affections as the one under consideration, in which but little progress has thus far been made, that a rich field is open for patient and careful labor.

Puerperal insanity may be sub-divided into two classes or types, viz.: the Melancholic which is comparatively rare; and the Maniacal which is more frequently met with, and it is to the latter form that attention is invited.

Puerperal mania is an affection which is of much more frequent occurrence than is at first supposed.

In the report of the hospital of Saltpetriere, it was found that one-twentieth of all the women admitted developed the affection, while among the more wealthy the proportion was placed at one-twelfth nearly. What the proportion of cases is in this country the writer has been unable to ascertain, but admitting that it is as great as in France, the grave nature of the affection renders necessary a more careful investigation of its causes and phenomena with a view to its more intelligent treatment and possible prevention.

Puerperal mania is to be distinguished from the temporary aberration sometimes occurring during pregnancy, labor and lactation the period of its onset being about the fifth day.

Many theories have been advanced as to the predisposing causes of this form of insanity, but as they have all failed, in any marked degree, to be sustained by investigation, but little weight can be attached to them. It has been noted, however, that in a certain proportion of cases there, existed a certain hereditary tendency to mental aberration; also that primipara are more liable than multipara; that exhausting labors also predispose to it; the sense of shame and degradation attending the confinement of an unmarried woman; and also confinement of a primipara taking place during advanced life. It is believed that an enfeebled condition of the system, by whatever cause produced, is one of the most important factors in its production. Blood-poisoning as a result of renal, disease has been strongly urged as an exciting cause by writers of authority, and especially by Simpson, but this theory has not been fully sustained by subsequent investigation, and may be viewed more in the light of a complication than in a causative relation.

The symptoms of this affection may be stated briefly as follows: The patient at first exhibits a restless, irritable and perhaps morose manner, gradually giving way to agitation and excitement, with an anxious and unpleasing countenance.

An intense dislike is apt to be manifested towards one or more near relatives, as, for instance, the husband or even the child. The patient refuses food; lochia entirely suppressed, as is generally also the case with the secretion of milk; urine markedly diminished; bowels constipated; skin hot and dry; face flushed and head hot; eyes staring and wild; and the pulse full and frequent. It is noticed also that patients laboring under this affection make use of the most immoral language, and, unless restrained, they are apt, in their furious delirium to do violence to their attendants, or the mania may even assume a suicidal tendency.

Patients generally labor under some strong delusion, and it has been noticed, as one of the peculiar characteristics of this form of insanity, that patients seem to be conscious that they are under the influence of a false belief.

As regards prognosis in these cases, two questions present themselves, viz: Will recovery take place? And if so, will there be a complete mental restoration?

An answer to the first involves a consideration of the general health of the patient, for the reason that it is believed to be a disease of exhaustion, and a favorable result can only be looked for in proportion as the patient can be sustained through this affection, which seems, as a result of observation, to have a definite duration. Any existing complications must, of necessity, be considered in their proper light when determining the question of ultimate recovery.

As regards circulation, Dr. Gaillard Thomas says "there is no doubt that one of the most important symptoms, as indicating the probability of a fatal result, is the extreme rapidity of the pulse." Much, of course, will also depend on the treatment, both medical and dietetic. When there are no complications, however, of a serious nature, the tendency on the part of the affection is toward recovery.

In this, the maniacal form of the puerperal insanity, there is more ground to hope for a complete restoration to reason, provided there is no hereditary tendency, than in the melancholic variety. The duration of acute mania is generally stated to be about three weeks, from which time there is a gradual restoration to reason.

Stripped of controversy and theorizing, the above is an outline of what is positively known concerning Puerperal Mania, and it will be at once seen that there is a broad field still open for investigation into its causes and treatment.

Some idea of the methods of treatment adopted by our allopathic brethren may be obtained from the following history:

Mrs, G., aet. 21, had always had tolerably good health ; confined for the first time April 10th ; labor natural ; female child, ordinary size and well developed. Case did well until April 14th, when she had a fainting spell, on recovering from which she was somewhat flighty. This condition continued to grow rapidly worse and on the following day (15th) had developed into a maniacal condition which was intermittent. The family physician, an allopath, was called in and administered opiates freely per rectum, by mouth, and hypodermically, but only to aggravate the delirium.

The lucid intervals gradually became less frequent and of shorter duration, until the mania became continuous. A number of remedies were tried and all signally failed, the delirium becoming of the most violent character necessitating the strapping of the patient to the bed to prevent self-mutilation and injury to the attendants. The patient continued to grow worse, and on the 19th the physicians, after a consultation, informed the family that they had exhausted their therapeutical resources and that the woman would probably not live more than twenty-four hours. The writer was called to the case on the afternoon of the 19th and found the patient with a violent mania, talking incoherently, and although naturally of a modest, retiring disposition, making use of the most obscene language, than which worse could not be imagined. Pulse 100; temperature in axilla 99.8. Bowels inclined to be loose and stools very offensive. Water passed four times daily, in napkins so that exact quantity could not be determined, but evidently sufficient, of a pale amber color, slightly acid reaction, specific gravity 1026, on standing deposited a thick whitish-yellow sediment consisting principally of urates, but repeated tests failed to give evidence of albumen. The surface of the body was warm without moisture; feet cold; face flushed; eyes staring and wild and pupils contracted.

An ice-bag had been kept applied to the head for two days, and doses of 80 drops of what was evidently a saturated (or nearly so) solution of Chloral Hydrate had been administered every three hours, without any benefit. Enemata of beef-tea, in conjunction with *Tinct. Opii.* had been given, to nourish patient. No subjective symptoms could be obtained, but there was evidently no pain, body well nourished and furnished no evidence of inflammatory or other morbid action on physical examination. The milk, which had been free, now was entirely suppressed, and mammæ very much reduced in size. Lochial discharge free, of a dark red color and very offensive.

The patient since the commencement of the attack, had not slept, except under the influence of chloroform. After as careful an examination as could be made under the circumstances the patient was placed on *Bell.* 1000th (Fincke) in water given at intervals of fifteen minutes; a hop-pillow substituted for the ice-bag; the vagina washed twice daily; and the patient nourished per orem with milk, strong beef-tea and gruel. The violence of the mania necesitated the continued use of the straps on the wrists and ankles, and body as well.

During the following night the patient slept about four hours, at intervals, next morning the symptoms were but little changed, the mania still violent, but with an occasional quiet interval of short duration; pulse 120; bowels moved twice during the night; water free with same properties as when first examined. During the day (April 20) the patient had a quiet, refreshing sleep of one hour, and all other symptoms began perceptibly to improve and, although the delirium still continued, it was less violent, and the calm periods more frequent, the incoherency giving place to more connected but at the same time very silly talk, accompanied with immoderate laughter. To-day, also for the first time, there was a partial recognition of members of the family. April 21st, the patient has so far improved in all of her symptoms that the *Bell.* is given at intervals of half an hour.

April 22d. Improvement still continues and medicine ordered to be given every hour; this was premature however, as during the following night the violence of the delirium returned, the bowels became constipated; urine was entirely suppressed; no sleep obtained; pulse which had fallen to 100 now rose to 120; face flushed and hot; eyes staring and pupils contracted. Medicine again given at quarter-hour intervals, when these symptoms rapidly subsided, and convalescence re-established. Throughout the affection the tongue had a brown heavy coating, the breath being fœtid. An abscess formed above Poupart's ligament midway between to Ant. Sup. Spinous process and the pubes on the left side, which on being opened discharged nearly a pint of laudable pus; a second abscess formed over the right Olecranon process which was also opened, and both were irrigated with Carbolized water, healing kindly and rapidly.

On April 29th, the patient's physical condition was such that, with the exception of a sense of weakness, felt during convalescence from any serious disorder, she was considered well, there being but an occasional forgetfulness or wandering of the mind, which in three weeks had almost entrely disappeared. At the fourth week the patient became unwell, and there was a return of all the more prominent symptoms of the previous attack but in a mild form which was controlled by *Bell.* The second attack, or relapse lasted but three or four days, disappearing gradually to return no more.

In this case there was no evidence of hereditary taint; cerebral congestion was excluded in the diagnosis from the entire absence of its characteristic phenomena; careful investigation of the urine failed to reveal the presence of albumen, and its absence, taken in connection with the large proportion of urates excreted, is sufficient proof of at least the usual activity of the kidneys.

If renal disease had existed, it had entirely disappeared when the writer first saw the case.

Excepting that the patient was a primipara, and far from being robust, it is a noticeable fact, that all those causes, whether predisposing or exciting, to which this affection has usually been ascribed, were entirely wanting in this case.

The abscesses mentioned were of slow formation and, as they were very superficial (being situated in the subcutaneous areolar tissue, and not connected by sinuses with any of the deeper structures), have been considered as complications and treated accordingly with very satisfactory results.

Although nothing absolutely certain could be determined as to causation in this case, the writer believes that two influences at least were responsible for its appearance, viz: 1st, general debility and 2d, a condition of toxæmia.

The health of the patient, while it had been fair and free from any organic disease, during pregnancy, was anything but vigorous and her general appearance that of anæmia.

The consideration of the second and more important of the causes forms a subject upon which much might be said, and opens up an extensive field for investigation, and, as it would be impossible to do it justice in this article, I shall merely state my reasons for the opinion I entertain, namely, that the mania is due to blood poisoning as the result of the accumulation in that fluid of the products of involution of the uterus.

My reasons for this are briefly as follows, viz:

First. The time of its appearance coincides with the period at which physiologists inform us the uterus has sensibly advanced in the process of involution, *i. e.*, from the fourth to the sixth day.

Second. The general symptoms of toxæmia.

Third. The recurrence of all the symptoms, though greatly diminished in severity, with the return of the menses, when the thickened mucous membrane is undergoing the physiological changes incident to that period.

Fourth. The presence of a large amount of uric acid (urates) in the urine whose formation is supposed to be caused by the imperfect oxidation of the nitrogenous principles resulting from disassimilation. This would seem to be supported by the belief that, during involution after parturition, and also during menstruation, there exists in the blood an increased amount of excrementitious nitrogenous matter, as a result of these physiological processes, and this taken in conjunction with the diminution of the quantity of red corpuscles of the blood in anæmia would account for its imperfect oxidation.

Without attempting to elaborate, I have presented, as briefly as possible views concerning the causes of this affection, hoping, if nothing more, it may stimulate more earnest investigation and result in positively determining its etiology.

